# In vitro anticancer potential of laminarin and fucoidan from Brown seaweeds

**DOI:** 10.1038/s41598-023-41327-7

**Published:** 2023-09-02

**Authors:** Elumalai Sanniyasi, Rajesh Kanna Gopal, Rajesh Damodharan, Arthi Arumugam, Madhumitha Sampath Kumar, Nandhini Senthilkumar, Monisha Anbalagan

**Affiliations:** 1 Department of Biotechnology, University of Madras, Guindy Campus, Chennai, 600025 India; 2 Department of Microbiology, Saveetha Dental College and Hospitals, SIMATS, Chennai, 600077 India; 3 Department of Biotechnology, Rajalakshmi Engineering College, Chennai, 602105 India; 4 Department of Biotechnology, Jeppiar Engineering College, Chennai, 600119 India

**Keywords:** Biotechnology, Cancer, Plant sciences

## Abstract

Marine seaweeds are rich source of polysaccharides present in their cell wall and are cultivated and consumed in China, Japan, Korea, and South Asian countries. Brown seaweeds (Phaeophyta) are rich source of polysaccharides such as Laminarin and Fucoidan. In present study, both the laminarin and fucoidan were isolated was yielded higher in PP (*Padina pavonica*) (4.36%) and STM (*Stoechospermum marginatum*) (2.32%), respectively. The carbohydrate content in laminarin and fucoidan was 86.91% and 87.36%, whereas the sulphate content in fucoidan was 20.68%. Glucose and mannose were the major monosaccharide units in laminarin (PP), however, fucose, galactose, and xylose in fucoidan (STM). FT-IR down peaks represent the carbohydrate of laminarin and fucoidan except, for 1219 cm^−1^, and 843 cm^−1^, illustrating the sulphate groups of fucoidan. The molecular weight of laminarin was 3–5 kDa, and the same for fucoidan was 2–6 kDa, respectively. Both the Fucoidan and Laminarin showed null cytotoxicity on Vero cells. Contrastingly, the fucoidan possess cytotoxic activity on human liver cancer cells (HepG2) (IC_50_—24.4 ± 1.5 µg/mL). Simultaneously, laminarin also shown cytotoxicity on human colon cancer cells (HT-29) (IC_50_—57 ± 1.2 µg/mL). The AO/EB (Acriding Orange/Ethidium Bromide) assay significantly resulted in apoptosis and necrosis upon laminarin and fucoidan treatments, respectively. The DNA fragmentation results support necrotic cancer cell death. Therefore, laminarin and fucoidan from PP and STM were potential bioactive compounds for anticancer therapy.

## Introduction

Our Mother Earth serves us enumerable natural resources for our daily needs and it is composed of 70% of the ocean and scavenges 50% of carbon dioxide (CO_2_) from the atmospheric air and provides about half of the Earth’s Oxygen (O_2_). Marine is a major and gifted natural resource for a nation with sea borders. Biotic resources of the marine ecosystems include Phytoplanktons (Microalgae), Zooplanktons, Fishes (including mammals like Whales and Dolphins), Seaweeds, Sea Grass, Sea Anemones, Crabs, Corals, Sea Sponges, Sea Snails, Sea Urchin, Sea Turtles, Sea Cucumber, and Star Fish. Despite this, 95% of the Ocean is still unexplored.

With only 2–4% of the Globe’s land mass, India has 7–8% of the World’s species diversity including 45,000 species of plants and double the number of animals. Thus, India is described as the 12th Megadiverse nation in the world. India is bordered by the Bay of Bengal in the East, the Arabian Sea in the West, and the Indian Ocean in the South, and hence rich in vast Marine Resources. Seaweeds are eukaryotic and thallophytic macroalgae classified into Phaeophyceae (Brown Algae), Rhodophyceae (Red Algae), and Chlorophyceae (Green Algae). India is rich in 841 species of Seaweeds, including 434 species of Rhodophyceae, 191 species of Phaeophyceae, and 216 species of Chlorophyceae^1^. The Gulf of Kutch in Gujarat and the Gulf of Mannar in Tamil Nadu are seaweed diversity-rich regions in India^2^. About 80 species of seaweeds were reported from the Andaman and Nicobar Islands^3^. The Gulf of Kutch engulfs about 198 species of seaweeds including 109 species of Rhodophyceae, 54 species of Chlorophyceae, and 35 species of Phaeophyceae^4^. However, in the Gulf of Mannar, 282 species of seaweeds with 146 Rhodophyceae, 80 Chlorophyceae, and 56 Phaeophyceae were reported^2^.

Seaweeds are rich sources of cell wall polysaccharides commercially termed Phycocolloids, which are large molecular weight and non-crystalline substances. Three major Industrial Phycocolloids are Alginate, Agar, and Carrageenan. Alginate is derived from Brown seaweeds (Phaeophyceae), whereas, Agar and Carrageenan are extracted and purified from Red seaweeds (Rhodophyceae).

Laminarin is also a short-chain, non-sulphated, Glucan rich cell wall polysaccharide reported with Anticancer^5–9^, Antioxidant^10^, Anti-inflammatory^11,12^, and Antiviral activities^13,14^. The dietary β-glucan is reported with hypocholesterolemic effect and minimizing heart disease^15^, and prevents breast cancer in human^16^. Laminarin is a β-glucan from Brown seaweeds have a linear backbone linked by 1-3 β-glycosidic bonds and low molecular weight of approximately 5 kDa^17^. However, the structure and function of laminarin from different Brown seaweeds may vary in their bioactive potential, but it was reported to possess anticancer activities on wide range of cancer cell lines such as LoVo (Colorectal cancer)^5,18^, HT-29 (Melanoma)^19^, HCT 116, SK-MEL-5, MDA-MB-231 cell lines (Breast cancer)^20^.

Unlike laminarin, fucoidan are rich in sulphated fucans dispersed the cell walls of Brown seaweeds. Fucoidan is a long-chain, sulphated, fucose rich polysaccharide found in the cell walls of Phaeophyceae (Brown algae) reported with several bioactive properties such as Anticancer^21,22^, Antiviral^23,24^, Anti-inflammatory^25^, Immunomodulatory^26^, Prebiotic^27^, Wound healing^28^, Chronic anti-renal failure^29^, Anti-ulcer activities (*Helicobacter pylori*)^30^. Fucoidan from *Fucus vesiculosus* enhanced apoptotic activity in 4T1 breast cancer cells in vitro and in vivo^31^. However, in an interesting study, oral administration of seaweed powder (1.6 g kg^−1^ of body weight) from Brown seaweeds such as *Sargassum ringgoldianum, Laminaria japonica, Scytosiphon lomentaria*, and *Lessonia nigrescens* to in vivo mice model, significantly inhibits Ehrlich carcinoma by 46.5%, 57.6%, 69.8%, and 60%, respectively^32^. Simultaneously, fucoidan treatment on xenograft mouse models impressively suppressed tumor and metastasis effects^33,34^. Both laminarin and fucoidan extracted from *Laminaria japonica* were injected into mice and observed that the fucoidan showed strong activation of the immune system than laminarin and therefore, fucoidan enhanced the anticancer efficacy against Lewis lung carcinoma^35^. Fucoidan from *Turbinaria conoides*, had shown significant anticancer activity on MCF-7 (human breast cancer), and A549 (human lung cancer) cell lines, and no cytotoxic activity on L929 mouse fibroblast cell line^36^. Similarly, fucoidan from *Sargassim cinereum* inhibited the proliferation of CaCo-2 cell line (Colon cancer) on a dose-dependent manner^37^.

In the present study, four commonly available Brown seaweeds such as *Padina pavonica, Spatoglossum asperum, Sargassum wightii*, and *Stoechospermum marginatum* in the Mandapam coastal region were chosen for the study, and collected during the first week of March 2019 (Summer season) with a sea surface temperature ranges between 28 and 31 °C. However, July–January is the period of high yield for obtaining seaweed biomass and it is the optimal season for harvesting in the Gulf of Mannar coastal region^38^. There were so many reports on the anticancer activities of sulphated and non-sulphated polysaccharides from marine seaweeds. Hence, the novelty of the study is to assess the quantitative analysis of both laminarin (non-sulphated) and fucoidan (sulphated) from Brown seaweeds and their anticancer activity.

## Materials and methods

### Collection of marine macroalgae

Most commonly dispersed brown macroalgae including *Padina pavonica* (PP), *Spatoglossum asperum* (SPM), *Sargassam wightii* (SW), and *Stoechospermum marginatum* (STM) were collected from the Pudumadam, Mandapam Coastal region (East Coast) (9°16′21.67″ North, and 78°59′33.43″ East), Ramnad District, Tamil Nadu, India. The collected algal samples were morphologically identified by Dr. M. Balusami, former Professor, Department of Plant Biology and Plant Biotechnology, Madras Christian College (Autonomous), Chennai, Tamil Nadu, India. The collected macroalgae samples were washed thoroughly with tap water two or three times to remove epiphytes, salt, sand particles, shade dried, and grounded to fine powder for further extraction of crude polysaccharide.

### Extraction of polysaccharide from macroalgae

To a 100 g of finely grounded macroalgae biomass (PP, SPM, SW, and STM), 500 mL of distilled water (dH_2_O) was added and allowed to extract the polysaccharide by hot-water extraction method at 80 °C for 120 min. The extract was filtered through a nylon mesh and again the extraction procedure was repeated with adding additional 500 mL of dH_2_O. Then the filtered extract was pooled and centrifuged at 10,000 rpm for 5 min. The supernatant was reacted with four volumes of absolute ethanol and kept undisturbed overnight at 4 °C. Then the precipitated crude polysaccharide was segregated by centrifugation at 10,000 rpm for 5 min. and dried. Then the obtained pellet was diluted to a final volume of 1 L with dH_2_O and added with 2% Calcium chloride (CaCl_2_) to precipitate out alginate. After overnight incubation, the alginate content was pelleted out by centrifugation at 8000 rpm for 10 min. Then to the supernatant, again four volumes of absolute ethanol was added to precipitate out crude polysaccharide. Then the content was centrifuged at 10,000 rpm for 5 min. to yield polysaccharide. The pelleted crude polysaccharide content was dried using hot air oven at 40°C^23^.

### Purification of laminarin and fucoidan

Laminarin in a non-sulphated, charge less neutral polysaccharide, whereas, Fucoidan is a sulphated polysaccharide and it is negatively charged and thus, both the polysaccharides were purified by DEAE- cellulose by Anion-exchange chromatographic purification^23^. The obtained crude polysaccharide from PP, SPM, SW, and STM were dissolved in 10 mL of deionized water and kept ready for loading. Four 5 mL polypropylene syringes (1.2 mm diameter) were used as a column prepacked (1 cm gel bed) with DEAE-Cellulose (Sigma-Aldrich) for four different macroalgae samples PP, SPM, SW, and STM with a flow rate of 1.3 mL min^−1^. Further, the deionized water was used to wash the column and the elution were collected in separate tubes and dried to obtain purified Laminarin^39^. Then the total yield of laminarin was determined and compared among the four different macroalgae involved in this study.

After the segregation of laminarin, the negatively charged fucoidan bounded to the positively charged DEAE-Cellulose was released by using different gradient NaCl solutions ranging from 0 to 3.6 M concentrations with 0.4 M interval. The NaCl solutions were prepared in 50 mM sodium acetate buffer with pH of 5.0. Different molar NaCl elution were dialysed against deionized water using a membrane dialysis (Dialysis membrane-70, HI-MEDIA) for each macroalgae^23^. Then the different elution was subjected to drying and the obtained fucoidan were quantitatively determined by weighing and the total yield was compared among different macroalgae involved in this study^23^.

### Estimation of carbohydrate and sulphate

The total carbohydrate content was determined for both the laminarin and fucoidan samples based on the method described by Dubois et al.^40^. About 500 µL of the sample (after purification, and before drying) was allowed to react with 5% phenol and 2.5 mL of concentrated sulphuric acid. The mixture was kept undisturbed under incubation for 15 to 20 min at room temperature. The Absorbance values were estimated optically at 490 nm by using UV–Visible spectrophotometer. The carbohydrate content of samples was estimated by comparing the values with known standard. The d-glucose was used as standard.

The total sulphate content was determined based on the method described by Terho and Hartiala^41^. BaCl_2_ buffer solution was prepared by adding 10 mL of 2 M acetic acid with 2 mL of 0.005 M BaCl_2_, and 8 mL of 0.02 M NaHCO_3_, and made up the volume till 100 mL using absolute ethanol in a 100 mL standard flask. About 5 mg of Sodium rhodizonate was diluted in 20 mL of deionized water, and 100 mg of l-ascorbic acid was added and the volume was made up to 100 mL in a standard flask using absolute ethanol and the sodium rhodizonate solution was prepared 30 min prior to the assay. For standard graph, 2–12 µg of Na_2_SO_4_ was diluted in 0.5 mL of deionized water. To a 0.5 mL of standard, sample, Blank (0.5 mL of dH_2_O), 2 mL of absolute ethanol was added followed by the addition of 1 mL of BaCl_2_ buffer and 1.5 mL of sodium rhodizonate solution. All the tubes were mixed thoroughly and incubated in dark for 10 min at room temperature. Finally, the optical density was measured at 520 nm within 30 min. The quantity of sulphate content was determined by using the standard graph.

### Monosaccharide composition of laminarin and fucoidan

The purified laminarin and fucoidan (10 mg) were subjected to hydrolysis in 0.5 mL of 2 M trifluoro acetic acid (TFA) under 121 °C for an hour. After hydrolysis, the samples were derivatized with PMP (1-phenyl-3methyl-5-pyrazolone) by mixed with the same volume with 0.6 M NaOH and 100 µL of 0.5 M PMP diluted in methanol was added, vortexed, and kept under incubation for 1 h at 70 °C in a water bath. Then the cooled mixture was neutralized with 120 µL of 0.3 M HCl and dissolved in 1 mL of dH_2_O. A 1 mL of chloroform was added, vortexed and the obtained organic phase was discarded. Then the derivatized aqueous phase was filtered through 0.45 µm membrane filter and eluted with thermo hypersil ODS-2 C18 HPLC columns (250 mm × 4.6 mm with a flow rate of 1 mL min^−1^) at 30 °C. The UV absorbance of 245 nm was involved to detect the monosaccharide composition of laminarin and fucoidan^42^.

### Characterization of laminarin and fucoidan

#### Fourier transform infrared spectrometry (FT-IR) analysis

About 5 mg of laminarin and fucoidan samples were grounded separately with 100 mg of potassium bromide (KBr) each and compressed at 10,000 psi and the sample was ready for the FT-IR analysis. The FT-IR analysis (BRUKER MID-IR) was performed for the laminarin and fucoidan samples at a frequency ranging between 500 and 5000 cm^−1^^23^.

#### Matrix assisted laser desorption/ionization time of flight (MALDI-TOF) analysis

The molecular weight of both the laminarin and fucoidan were determined by the mass by charge ratio (*m/z*) analysed between 1000 and 10,000 m*/z* by MALDI-TOF analysis using MALDI-7090™ (SHIMADZU)^43^.

#### In vitro cytotoxic activity of laminarin and fucoidan

For cytotoxic activity assay, Vero cell line (obtained from National Center for Cell Scienes (NCCS), Pune, India) monolayer culture was trypsinized and the cells were counted and adjusted to 1 × 10^5^ cells mL^−1^ with the culture media having 10% FBS (Fetal Bovine Serum). Then 100 µL of suspended cells (1 × 10^4^ cells well^−1^) were seeded on to each wells of the microplate and incubated for 24 h with 5% CO_2_ in an incubator. After incubation, a monolayer was formed, the medium was discarded and the monolayer was washed with culture medium and added with 100 µL of different concentration of the test samples and again incubated at 37 °C for 24 h with 5% CO_2_ in an incubator. Then the next day, the test solutions in all the wells were discarded and 20 µL of MTT (2 mg mL^−1^ of MTT in PBS (Phosphate buffered saline) buffer) was added to all the wells, and again incubated for 4 h in an incubator with the above said conditions. After incubation, the supernatant was discarded and 100 µL of DMSO was added to each of the wells and the absorbance values were measured at 570 nm. The percentage of cell viability was calculated and recorded using the formula given below^44^.

$$\% \;{\text{ of}}\;{\text{ Cell}}\;{\text{ Viability}}\; \, = \;{\text{ ABS}}\;{\text{ value}}\;{\text{ of}}\;{\text{ sample }}/{\text{ ABS}}\;{\text{ value }}\;{\text{of }}\;{\text{control }} \times { 1}00$$

#### In vitro anticancer activity of laminarin and fucoidan

Two different cancer cell lines, such as Human Colon Cancer Cells (HT-29), and Human Liver Cancer Cells (HepG2) were chosen for Laminarin and Fucoidan, respectively to study their anticancer potential.

Both the Human Colon Cancer cell line (HT-29), and Human Liver Cancer cell line (HepG2) were obtained from the National Center for Cell Scienes (NCCS), Pune, India. The cell lines were separately sub-cultured and maintained in Dulbecco’s Modified Eagles Medium (DMEM) supplemented with Balanced Salt solution (BSS), and 2 mM l-glutamine along with 1.5 g L^−1^ of Na_2_CO_3_, 1 mM sodium pyruvate, 2 mM 1.5 g L^−1^ of glucose, 0.1 mM non-essential amino acids, 10% Fetal Bovine Serum (GIBCO, USA), and 10 mM HEPES buffer (4-(2-hydroxyethyl)-1-piperazineethane sulfonic acid). Finally, 1 mL L^−1^ of antibiotic (combination of Penicillin and Streptomycin (100 IU/100 µg)) was supplemented. Then both the HT-29 (Human Colon cancer cells), and HepG2 (Human Live cancer cells) were maintained at 37 °C using a humidified CO_2_ incubator supplemented with 5% CO_2_^45^.

The MTT assay is a colorimetric assay which depends on the metabolic activity of living cells, in which, the MTT [3-(4,5-dimethylthiazol-2-yl)-2,5-diphenyltetrazolium bromide] tetrazolium dye reduced to purple coloured formazan crystals when reacts with NADPH dependent oxidoreductase enzymes^46^. The same chemical reaction does not happen when the cells are dead and MTT remains idle. Both the cancer cell lines HT-29, and HepG2 were cultured in 96-well microplates (1 × 10^4^ cells well^−1^) for 48 h separately with modified DMEM. Then the medium was replaced with fresh medium consists of serially diluted test samples (1–100 µg mL^−1^ concentration with 20 mg mL^−1^ interval) (Laminarin for Human Colon cancer cells (HT-29) and Fucoidan for HepG2 (Human Liver cancer cells) and incubated for 24 h. Further, 100 µL of MTT (2 mg mL^−1^ of MTT in PBS buffer) was supplemented to each well and again incubated for 4 h at 37 °C in a CO_2_ incubator. About 50 µL of DMSO was added to each well and incubated for 10 min. to solubilize formazan crystals after removal of remaining medium from the wells. Finally, the optical density (OD) was measured at 570 nm using a microplate reader (Thermo Multiskan EX, USA). Percentage of viability was determined by the formula given below^46^.

$$\% \;{\text{ of}}\;{\text{ Cell}}\,{\text{ Viability }}\; = \, \;{\text{OD }}\;{\text{value }}\;{\text{of}}\;{\text{ experimental}}\;{\text{ sample}}/{\text{OD}}\;{\text{ value }}\;{\text{of }}\;{\text{experimental }}\,{\text{control }} \times { 1}00$$

Doxorubicin was used as a positive control and the inhibitory concentration (IC_50_) value was calculated and inferred. The in vitro studies were carried out in triplicates, and the SPSS version 17.0 was used for the statistical analysis. The *p*-value was found significant to < 0.05.

#### Acridine orange/ethidium bromide (AO/EB) staining assay

Acridine orange is a vital dye for staining and differentiate live and dead cells. And ethidium bromide binds only with the cells which lost membrane integrity and thus, live cells appear normally green in fluorescent microscope. Apoptotic dead cells incorporate acridine orange stain and looks orange in colour. Necrotic cells also stain in orange devoid of condensed chromatin, but resembles the nuclear morphology of living cells. In this present study, Both the control and test sample (laminarin and fucoidan) treated cancer cells (Human Colon cancer cells and Human Liver cancer cells) (3 × 10^4^ cells well^−1^) incubated for 24 h in a CO_2_ incubator were fixed in a glass slide with methanol and glacial acetic acid (3:1 ratio) solution for 30 min. at room temperature and washed with PBS buffer and then stained with 1:1 ratio of Acridine Orange / Ethidium bromide solution. Again, washed with PBS buffer and viewed and photographed under fluorescence microscope at 40× magnification (ECLIPSE, Nikon, Japan)^47^.

#### DNA fragmentation assay

The DNA fragmentation assay in agarose gel differentiates live and apoptotic cells, in which, a single DNA band shows live cells, whereas, dragged DNA bands like ladder represents the cells undergone apoptosis. The fragmentation of DNA is due to the activation of caspase activated DNase (CAD) that leads to apoptotic cancer cell death. Both the Human Colon cancer cells (HT-29), and Human Liver cancer cells (HepG2) (10^6^ cells well^−1^) treated with test samples Laminarin, and Fucoidan for 24 h were suspended in 10 mL of Tris–EDTA buffer (10 mM Tris HCl, and 10 mM EDTA) (pH 8.0). Then the cells were treated with proteinase K solution (2% SDS, and 20 mg mL^−1^ of Proteinase K in Tris–EDTA buffer) and kept for incubation at 37 °C for 3 h. Then the DNA segregated and extracted in alcohol solution constitutes Phenol, Chloroform, and Isoamyl alcohol (25:24:1 ratio). Then the extracted DNA was treated with DNase free RNase (20 mg mL^−1^) at 4 °C for 45 min. and precipitated with 1 mL of 2.5 M sodium acetate and washed twice with four volumes of absolute ethanol. The extracted DNA of control, and test sample treated cancer cells were electrophoresed in 2% agarose containing ethidium bromide (EtBr) and the gel was visualized in a Gel documentation system under UV trans-illumination and photographed^48^. This assay was outsourced and performed at the Greensmed Labs, Chennai, and received the results in March and April 2021. The gel images were sent as cropped and labelled with DNA bands. The complete gel image was not available or the file was expunged (Supplementary file).

## Results

### Collection of marine macroalgae

**Figure 1:**
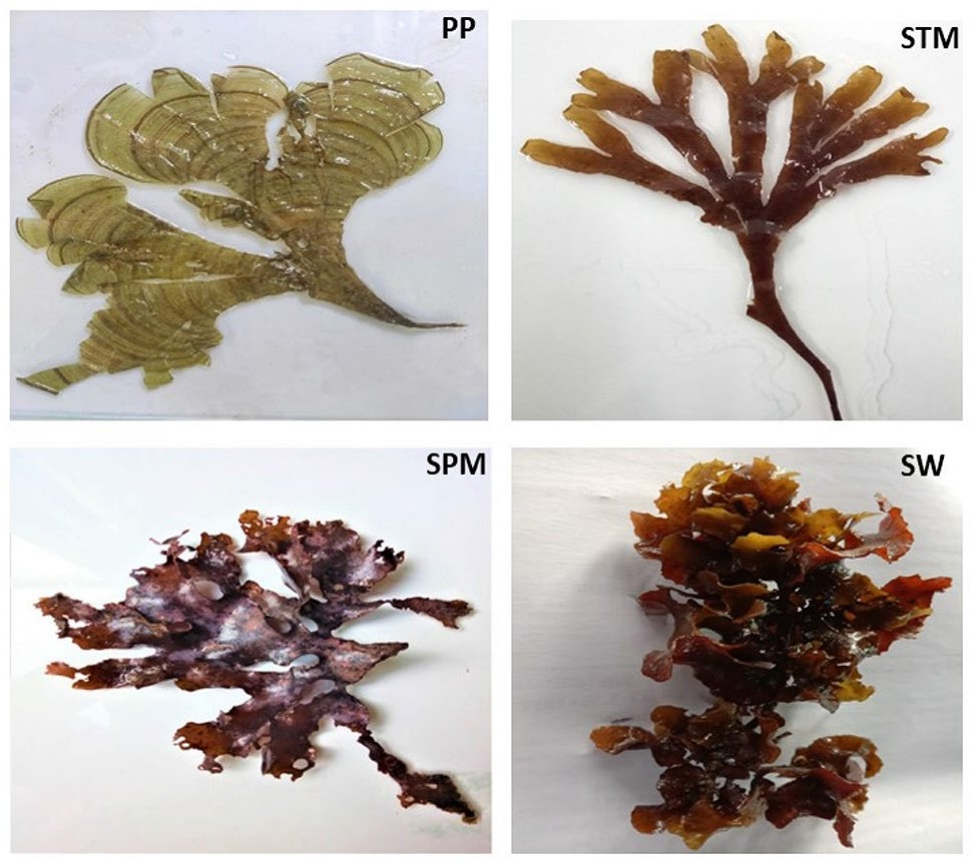
Photographical images of four different Brown seaweeds involved in the study *Padina pavonica* (PP); *Stoechospermum marginatum* (STM); *Spatoglossum asperum* (SPM); *Sargassum wightii* (SW).

Four different seaweeds belong to Phaeophyceae including *Padina pavonica* (PP), *Stoechospermum marginatum* (STM), *Spatoglossum asperum* (SPM), and *Sargassam wightii* (SW) were collected from the Mandapam Coastal region of Ramnad District, Tamil Nadu during January 2021 (Fig. 1).

### Extraction of laminarin and fucoidan from Brown seaweeds

Finely powdered seaweed biomasses were subjected to hot-water extraction at 70 °C for 2 h and 30 min. Alginate was removed by adding 2% calcium chloride solution by precipitation. Finally, crude laminarin and fucoidan were obtained by adding equal volumes of ethanol and centrifugation at 8000 rpm for 5 min. Laminarin is a neutral polysaccharide it was purified by using anion exchange chromatography. The percentage yield of laminarin obtained was 4.36% in PP, 1.86% in SPM, 0.60% in SW and 0.27% in STM (Table 1). Among different molar concentrations of NaCl, 0.8 M was found effective in eluting fucoidan in all the seaweed samples. Again, the elutions were dialysed against Milli-Q water and treated with equal volumes of ethanol to precipitate fucoidan and yielded by centrifugation at 10,000 rpm for 10 min. Among four different marine algae involved in our study, STM have yielded high amount of fucoidan with 2.32%, and SW ranked second with total fucoidan yield of 1.45%, but it was low in PP (0.68%) and SPM (0.53%) (Table 1).

**Table 1:** The table illustrates the yield of laminarin and fucoidan, and carbohydrate content of laminarin and fucoidan, and sulphate content of fucoidan.

Seaweed	Total laminarin yield (%)	Carbohydrate in Laminarin (mg/60 mg)	Total fucoidan yield (%)	Carbohydrate in Fucoidan (mg/60 mg)	Sulphate content in Fucoidan (%)
PP	4.36	52.15 ± 0.26 (86.91%)	0.68	31.9 ± 1.23 (53.16%)	23.52
SPM	1.86	32.07 ± 2.24 (53.45%)	0.53	28.74 ± 0.35 (47.9%)	18.86
SW	0.60	41.39 ± 0.60 (68.9%)	1.45	40.68 ± 1.07 (67.66%)	17.93
STM	0.27	45.13 ± 1.49 (75.21%)	2.32	52.42 ± 1.26 (87.36%)	20.68

### Estimation of carbohydrate and sulphate

Laminarin carbohydrate content was found higher in both the PP 52.15 ± 0.26 mg/60 mg (86.91%) and STM 45.13 ± 1.49 mg/60 mg (75.21%), and lower in SW (41.39 ± 0.060 mg/60 mg) (68.9%) and SPM (32.07 ± 2.24 mg/60 mg) (53.45%) (Table 1). However, the carbohydrate content in fucoidan was found greater in STM (52.42 ± 1.26 mg/60 mg) (87.36%) and SW (40.68 ± 1.07 mg/60 mg) (67.66%) and lesser in PP (31.9 ± 1.23 mg/60 mg) (53.16%) and SPM (28.74 ± 0.35 mg/60 mg) (47.9%) (Table 1). Among four different fucoidan samples, sulphate content was 23.52% in PP, 20.68% in STM, 17.93% in SW, and 18.86 in SPM (Table 1). The laminarin to fucoidan ratio was 85:15 and 76:24 in seaweeds PP and SPM, whereas, the same was 30:70, and 10:90 in SW and STM.

### Monosaccharide composition of laminarin and fucoidan

The laminarin constitutes of greater percentage of glucose (79.4%), with least percentage of mannose (13.2%), ribose (1.4%), arabinose (2.5%), xylose (0.7%), and galactose (0.5%), whereas, fucoidan constitutes of 64.2% of fucose, followed by low percentage concentration of galactose (14.5%), xylose (12.9%), mannose (4.3%), and rhamnose (3.2%) (Table 2).

**Table 2:** Monosaccharide composition of laminarin and fucoidan derived from PP and STM.

Polysaccharide sample	Monosaccharide units	Percentage (%)
Laminarin		
	Glucose	79.4
	Mannose	13.2
	Ribose	1.4
	Arabinose	2.5
	Xylose	0.7
	Galactose	0.5
Fucoidan		
	Fucose	64.2
	Galactose	14.5
	Xylose	12.9
	Mannose	4.3
	Rhamnose	3.2

### Characterization ofl laminarin and fucoidan

#### Fourier transform infrared spectroscopy (FT-IR)

**Figure 2:**
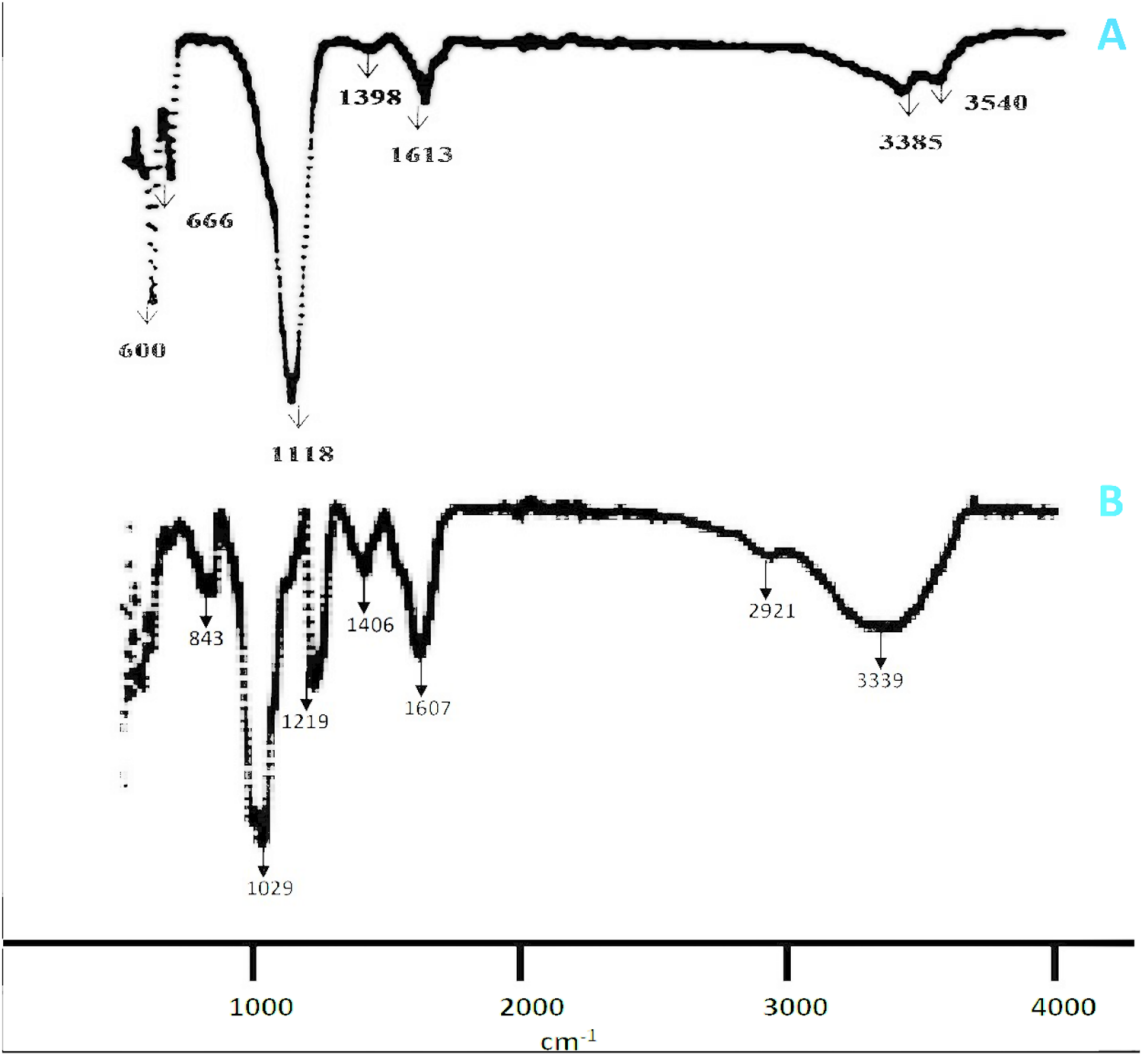
(**A**) FTIR chromatogram of laminarin; (**B**) fucoidan.

The down peak frequency wavelengths 3540 cm^−1^, 3385 cm^−1^, 1613 cm^−1^, 1398 cm^−1^, 1118 cm^−1^, 666 cm^−1^, and 600 cm^−1^ in laminarin and 3339 cm^−1^, 2921 cm^−1^, 1607 cm^−1^, 1406 cm^−2^, and 1029 cm^−1^ in fucoidan represents the hydroxyl groups, CH stretch, C–C (in-ring) stretches of the polysaccharides, whereas, 1219 cm^−1^, and 843 cm^−1^ obtained only from fucoidan represents the sulphate groups (Table 3 and Fig. 2).

**Table 3:** Table shows the IR peaks at certain frequency represent the respective functional groups obtained from laminarin (PP) and fucoidan (STM).

Laminarin (PP) Frequency (cm^−1^)	Fucoidan (STM) Frequency (cm^−1^)	Functional groups
3540	3339	OH Stretch, Hydroxyl group
3385		OH Stretch, Hydroxyl group
	2921	CH Stretch
1613	1607	C–C Stretch (in-ring)
1398	1406	C–C Stretch (in-ring)
	**1219**	**R-SO**_**3**_^**−**^** Sulfonates**
1118	1029	C–H Wag (–CH2X)
	**843**	**CO-S Stretch of sulphate group**
666		C–H bend
600		C–H bend

Significant values in bold represents the sulphate groups of fucoidan.

### Matrix assisted laser desorption/ionization-time of flight (MALDI-TOF) analysis of laminarin from PP and fucoidan from STM

**Figure 3:**
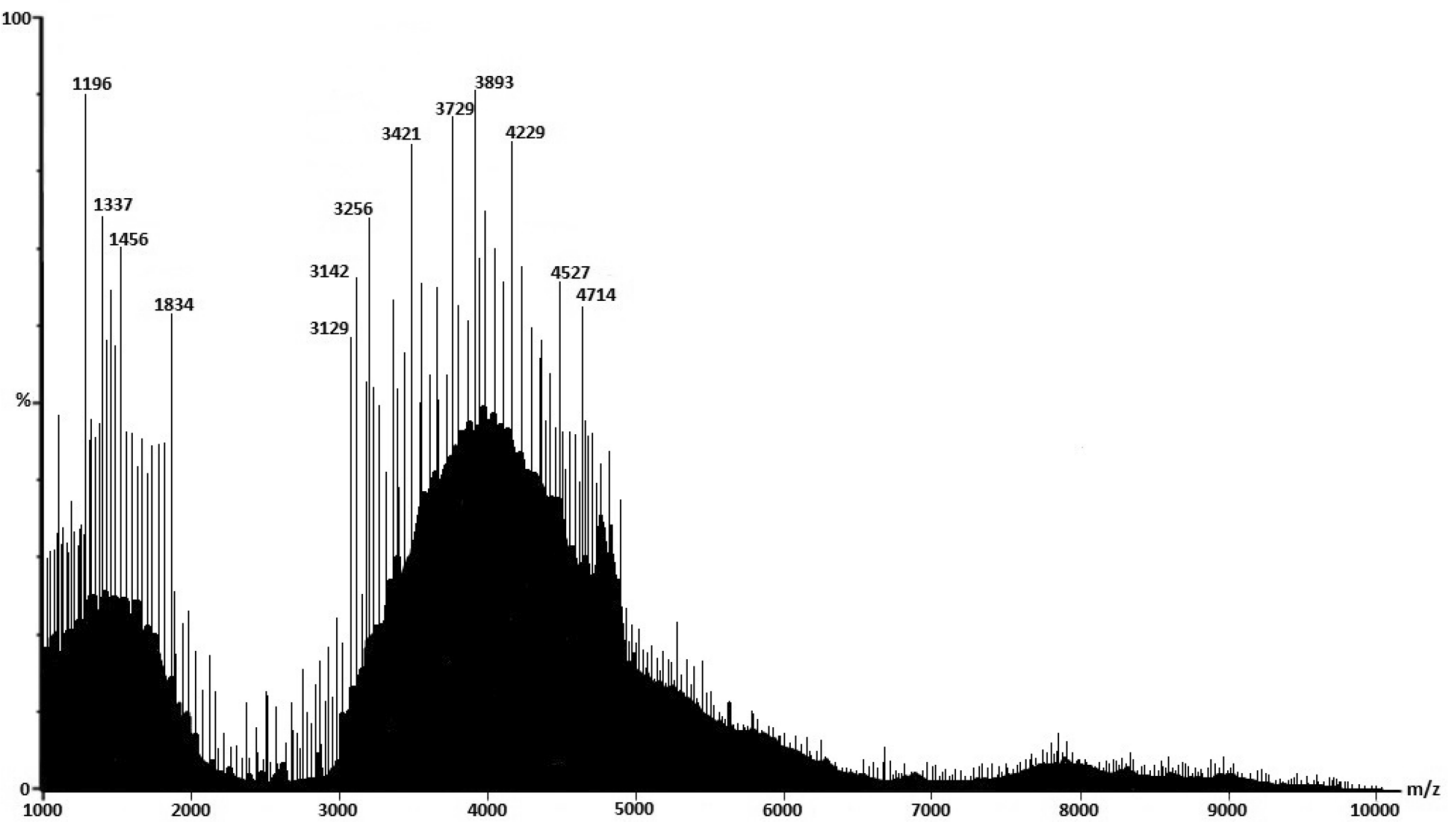
MALDI-TOF chromatogram of laminarin from PP.

**Figure 4:**
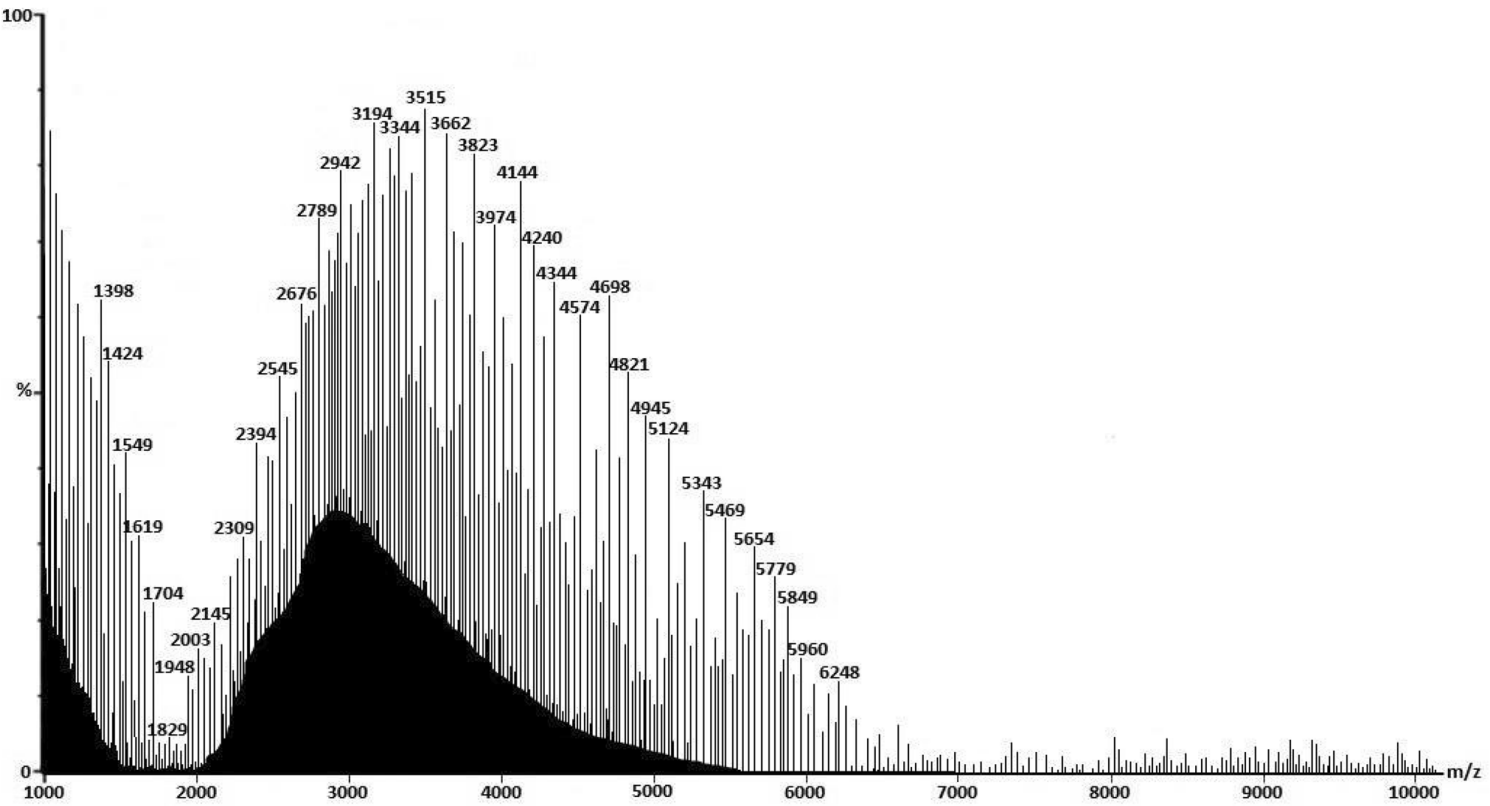
MALDI-TOF chromatogram of fucoidan from STM.

Based on the results obtained from MALDI-TOF, the *m/z* between 3000 and 5000 represents low molecular weight Laminarin ranging from 3 kDa daltons to 5 kDa daltons in *Padina pavonica* (PP) (Fig. 3). Similarly, peaks from 2000 to 6000 m*/z* represents low molecular weight fucoidan ranges between 2 and 6 kDa (Fig. 4).

### In vitro cytotoxic activity of laminarin and fucoidan

**Figure 5:**
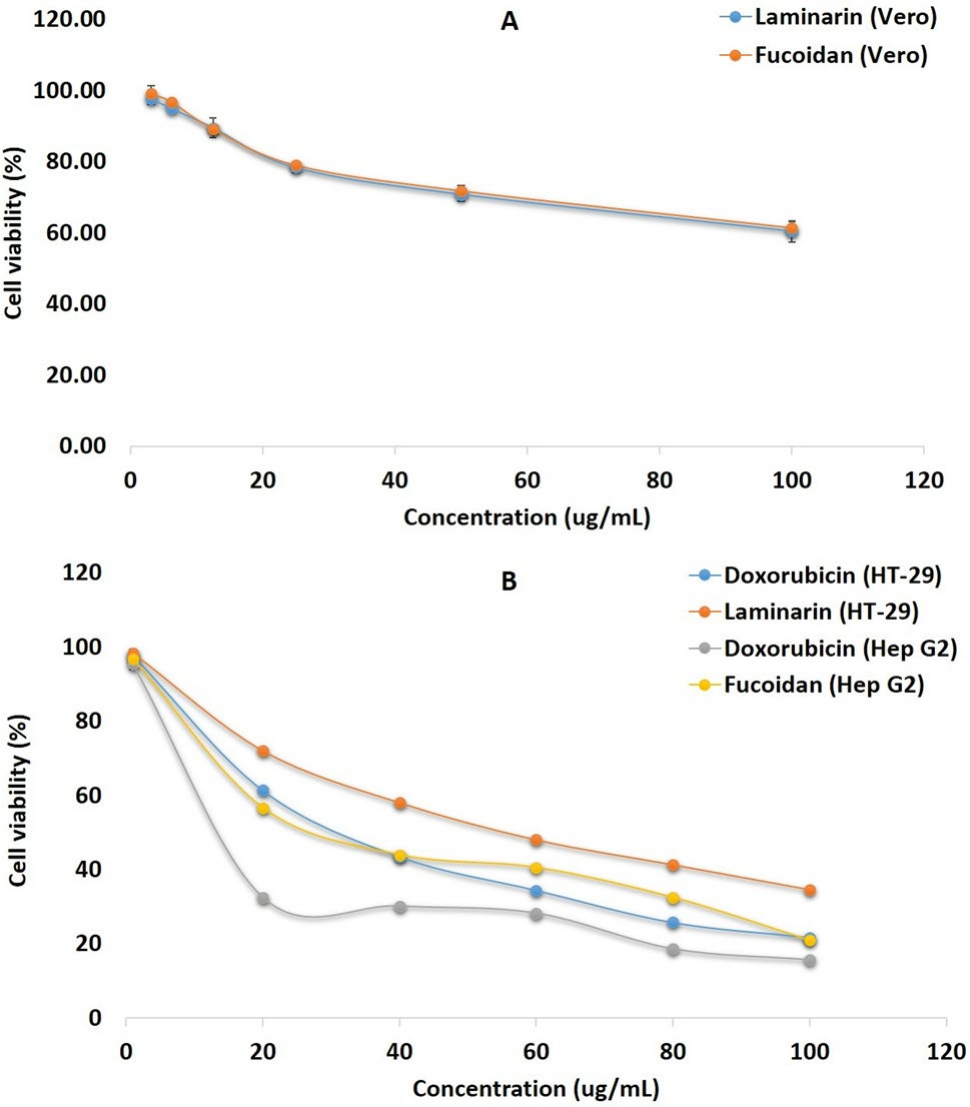
(**A**) MTT cytotoxicity assay of laminarin and fucoidan on Vero cell line; (**B**) MTT cytotoxicity assay of laminarin and fucoidan on HT-29 human colon cancer and Hep G2 human liver cancer cell lines, respectively.

Both the laminarin and fucoidan showed no cytotoxic activity on Vero cells with IC_50_ values of 123.54 ± 0.8 µg mL^−1^ and 114.89 ± 1.3 µg mL^−1^, respectively (Fig. 5A).

### In vitro anticancer activity of laminarin and fucoidan

#### MTT cell cytotoxicity assay

Based on MTT cell cytotoxicity assay, the laminarin purified from PP was found effective with potent anticancer activity. Figure 5B showing that the cell viability of colon cancer cells (HT-29) were found suppressed by increasing the concentration of laminarin in comparison with the positive control Doxorubicin. Finally, the IC_50_ (Inhibitory Concentration) value of laminarin was determined as 57 ± 1.2 µg mL^−1^, whereas, the IC_50_ value of standard was 31 ± 0.5 µg mL^−1^.

Similarly, the fucoidan extracted and purified from the marine brown alga STM possess effective anticancer activity showing anti-proliferative action on human liver cancer cells (Hep G2). It is clear that while increasing the concentration of the test compound (Fucoidan) percentage of cancer cell proliferation rate decreases consistently and found comparable with that of the standard Doxorubicin (Fig. 5B). The IC_50_ values determined were 24.4 ± 1.5 µg mL^−1^ and 5.4 ± 0.5 µg mL^−1^ for fucoidan and standard, respectively.

#### Acridine orange/ethidium bromide (AO/EB) staining assay

**Figure 6:**
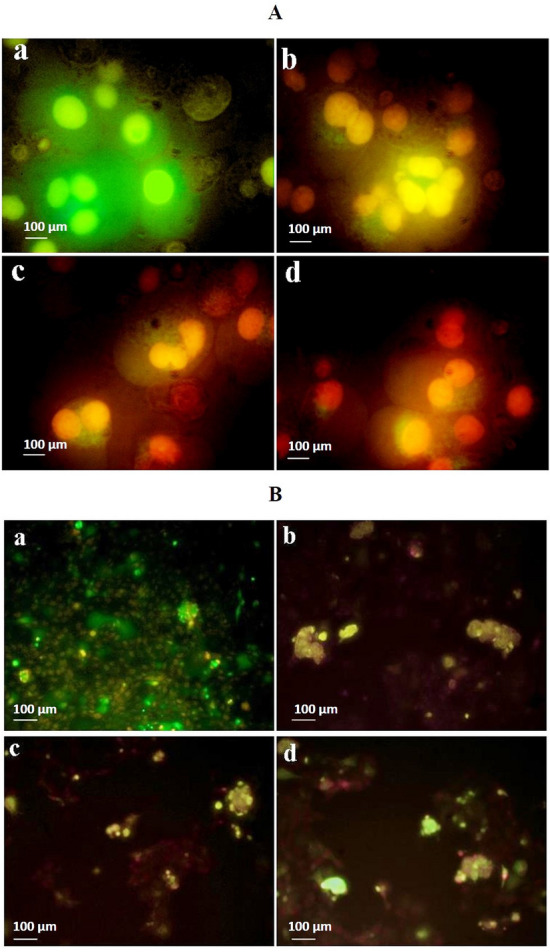
Acridine orange staining assay showing necrosis of test compound (**A)** Laminarin on Human colon cancer cells (HT-29); and (**B)** Fucoidan on Human liver cancer cells (Hep G2). (a) Negative control (no treatment), (b) 10 µg mL^−1^ of test sample, (c) 25 µg mL^−1^ of test sample, and (d) 50 µg mL^−1^ of test sample.

The treatment of test compounds Laminarin from PP and Fucoidan from STM at different concentrations (10 µg mL^−1^, 25 µg mL^−1^, 50 µg mL^−1^) with Human colon cancer (HT-29) and liver cancer (Hep G2) had resulted that the dead and live cancer cells were clearly discriminated by staining and observed under fluorescent microscopy (Fig. 6A and B). The orange and red fluorescensce in laminarin treatment on HT-29 cell line, and red fluorescence on fucoidan treatment against Hep G2 cell line clearly indicates the apoptoric and necrotic death phases, respectively.

#### DNA fragmentation assay

**Figure 7:**
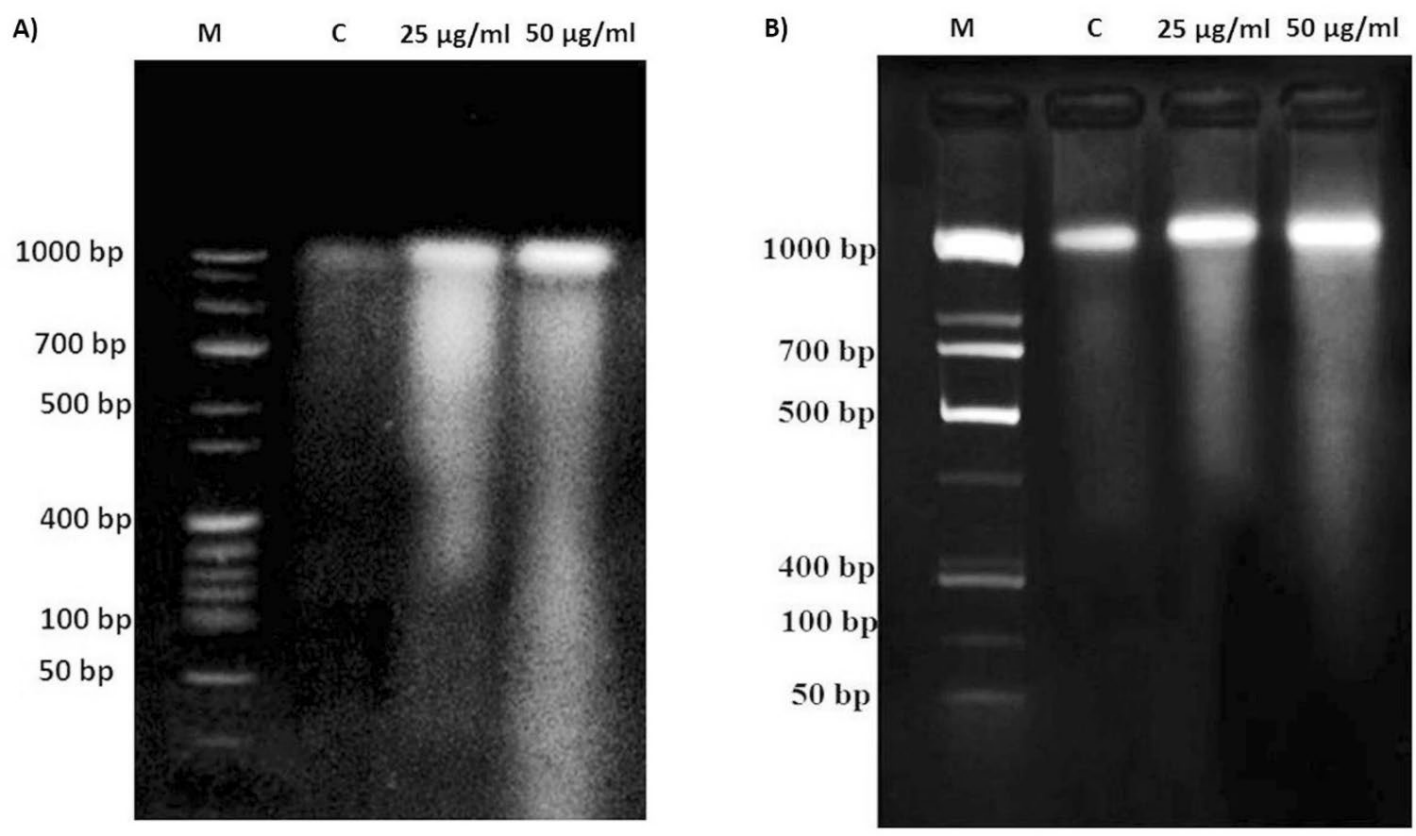
DNA fragmentaion assay showing necrotic activity of the test compounds (**A**) Laminarin from PP on human colon cancer cells (HT-29) and (**B**) Fucoidan from STM on human liver cancer cells (Hep G2); Lane 1: DNA marker; Lane 2: Negative control; Lane 3: 25 µg mL^−1^ of test compound; and Lane 4: 50 µg mL^−1^ of test compound.

The DNA fragmentation assay clearly resulted the anticancer activity of the test compound laminarin from PP and fucoidan from STM. The different concentrations of laminarin and fucoidan (25 µg mL^−1^ and 50 µg mL^−1^) showed DNA smear by forming sheared DNA fragments of dead human colon cancer cells (HT-29) and liver cancer cells (Hep G2), respectively (Fig. 7). Thus, it is evidently clear that both the laminarin and fucoidan exhibits necrotic activity on cancer cells.

## Discussion

For the past three decades, seaweeds contributed to many pharmaceutical applications on gall stones, renal disorders, cancer, heart disease, asthma, psoriasis, antibacterial, antifungal, antiviral, and arthritis with a wide range of metabolites like polysaccharides, phlorotannins, glycoproteins, terpenoids, alkaloids, lectins, pigments, and ketones^49^. Moreover, Fucoidan has a potent antiviral activity on SARS-CoV-2 (COVID-19) viral infection^50–53^. Simultaneously, laminarin also has potential antiviral activity on HIV^13^, reported to possess prebiotic activity on microbial gut health^54,55^, and antitumor activity^56^.

*Padina pavonica*, *Stoechospermum marginatum*, *Spatoglossum asperum*, and *Sargassum wightii* are the most frequently occurring Brown seaweeds in the Gulf of Mannar, Rameswaram, Tamil Nadu, India. In this present study, the four different seaweeds were collected from the Mandapam Coastal region, Rameswaram, Tamil Nadu, India followed by the extraction, purification, and evaluation of yield obtained for both Fucoidan and Laminarin. Both the polysaccharides were extracted using high polar solvent water and aqueous extraction by heat (Hot-water extraction method) was found cost-effective and the potential extraction method optimized. When treating crude extract with calcium ions (CaCl_2_), alginate gets precipitated and separated by centrifugation^57^. Hence, 2% CaCl_2_ was used in this study, alginate was separated and, finally, a crude extract containing fucoidan and laminarin was precipitated by adding equal amount of ethanol.

In present study, the obtained total laminarin and fucoidan yield was higher in *Padina pavonica* (4.36%) and *Stoechospermum marginatum* (2.32%), respectively, whereas, the fucoidan yield was 9.46% in *P. tetrastromatica*, 5.83% in *Turbinaria ornata*, 3.90% in *Sargassum wightii*^58^; 16% in *Laminaria japonica*. However, in another study, laminarin yield was 32% in diatoms *Skeletonema costatum*, 14% in *Phaeodactylum tricornutum*; 0.4% in *Stephanodiscus meyerii* and 15% in *Odontella aurita*^59^; 3–6% in seaweed *Laminaria hyperborea* and 4–5% in *Ascophyllum nodosum*^17^. The total laminarin content as storage carbohydrates in macroalgae was 1– 25% as dry weight^60^.

The estimated carbohydrate content in Laminarin was found higher in *Padina pavonica* with 52.15 ± 0.26 mg/60 mg (86.91%), whereas for fucoidan it was 52.42 ± 1.26 mg/60 mg (87.36%) in *Stoechospermum marginatum*. Similarly, sulphate content of fucoidan from *Stoechospermum marginatum* was 20.68%. Ohlsson et al.^61^ reported that the seaweed biomass is rich in 90% of carbohydrate, in which 60% of were laminarin from *Laminaria digitata*. The total carbohydrate yield was 10–11% from the seaweed biomass^58^. In another study the total fucoidan content obtained from *Turbinaria decurrens* and *Dictyota bartayresiana* were 3.89% and 8.67%, respectively^23^. Whereas, the range of suphate groups in fucoidan was between 5 and 40% in *Sargassum swartzii*^62^. However, fucoidan consists of 55% of carbohydrate and 40% of sulphate in its constituent^63^. Intriguingly, the carbohydrate content was greater during Summer^60^. Becker et al.^64^ resulted about 53% of carbohydrate from the biomass and among them, 42% were laminarin in diatom.

The monosaccharide composition of laminarin constitutes of 46.93% of fucose, 12.12% of galactose, 8.61% of arabinose, 4.23% of glucose, and 3.27% of mannose^12^. However, glucose alone found reported in the laminarin^65^. Concurrently, in the present investigation, laminarin derived from *Padina pavonica* constitutes of 79.4% of glucose, 13.2% of mannose. 1.4% of ribose, 2.5% of arabinose, and 0.7% of xylose and 0.5% of galactose. Xia et al. reported that the laminarin isolated from a marine diatom *Oduntella aurita* consists of about 82.23% of glucose units^66^. In the case fucoidan, fucose constitutes 64.2%, galactose 14.5%, xylose 12.9%, mannose 4.3%, and rhamnose 3.2% in *Stoechosermum marginatum* (present study), whereas, fucoidan from *Fucus vesiculosus* constitutes of 59.2% of fucose, 12.6% of xylose, 2.7% of mannose, 10.4% of galactose, 1.4% of rhamnose, and 13.6% of glucose^67^. The monosaccharide units of fucoidan from *Turbinaria decurrens* were fucose (59.3%), galactose (12.6%), mannose (9.6%), and rhamnose (6.4%)^68^. Similarly, fucoidan from *Fucus serratus* reported 40.6% of fucose, 43.2% of glucose, 10.4% of xylose, 4.2% of galactose, and 2.7% of mannose^67^. About 96.1% of fucose, and 3.9% of galactose were reported from *Fucus evascens*, and 38.7% of fucose, 32% of xylose, 16.2% of galactose, 5.6% of mannose, 2.3% of rhamnose, and 5% of glucose from *Dictyosiphon foeniculaceus*^67^. Simultaneously, fucoidan purified from *Laminaria digitata* constitutes 67.1% of fucose, 13.7% of xylose, 14% of galactose, and 5.2% of mannose. However, fucoidan from *Saccharima latissima* reported with 83.8% of fucose, 7.5% of galactose, 6.7% of xylose, and 2.1% of rhamnose^67^. Therefore, it has been determined that the glucose, and fucose are the major backbone monosaccharide unit of laminarin and fucoidan, respectively.

The FT-IR analysis resulted that the functional groups represent the carbohydrate nature of laminarin (non-sulphated polysaccharide), and carbohydrate and sulphate nature of fucoidan (Sulphated polysaccharide) from *Padina pavonica*, and *Stoechospermum marginatum* respectivley. The presence of sulphate groups in Fucoidan was clearly seen at 1219 cm^−1^, and 843 cm^−1^ respectivley. Congruently, 843 cm^−1^, 842 cm^−1^, 842 cm^−1^, and 820 cm^−1^ representing sulphate groups of fucoidan were reported from the respective marine seaweeds, which are *Padina tetrastromatica*, *Sargassum oligocystum*, *Laminaria Cichorioides*, *Fucus evanescens*, and *Laminaria japonica*^58,69^. In another study, 1200 cm^−1^, and 1201 cm^−1^ represents sulphonate groups of Fucoidan^23^ and also in our study the same results were seen at 1219 cm^−1^. This similar kind of FT-IR peaks were not seen in the Laminarin of *Padina pavonica* and hence it is devoid of sulphate group. In laminarin, 3400 cm^−1^ represents the hydroxyl group (–OH)^70^, whereas, hydroxyl groups were seen at 3540 cm^−1^, and 3385 cm^−1^ in the laminarin from *Padina pavonica*. Similarly, pure laminarin exhibited anisomeric stretching at 1620 cm^−1^^71^, in our present study the same was obtained at 1613 cm^−1^ in the laminarin. Carboxylic group of laminarin was obtained at 1398 cm^−1^ in laminarin of *Padina pavonica*, whereas it was reported in the laminarin at 1420 cm^−1^ from *Laminaria japonica*^72^. The presence of CC stretch (glycosidinc bonds) was determined at 1100 cm^−1^ in laminarin, and also the obtained result in this study as laminarin of *Padina pavonica* at 1118 cm^−1^^73^. However, the fucoidan from *Stoechospermum marginatum* was hydrolyzed to retrieve Fucose and determined by RP-HPLC with a Standard Fucose (Sigma-Aldrich). Hence, it is confirmed that the extracted sulphated polysaccharide from *Stoechospermum marginatum* was Fucoidan.

The obtained mass of laminarin from *Padina pavonica* was 3000 to 5000 daltons, whereas, for fucoidan from *Stoechospermum marginatum*, it was between 2000 and 6000 daltons. About 2–7 kDa molecular mass of Laminarin was determined from *Sargassum cichorioides*, and *Laminaria gurjanovae,* respectively^74^. Graiff et al.^43^ also determined the molecular mass of laminarin ranges between 2 and 7 kDa from *Lamanaria digitata*. The average molecular mass of laminarin was approximately 5 kDa^75–77^. High molecular weight fucoidan was reported from *Saragassum fusiforme* at an average weight of 95 kDa^78^. Similarly, high molecular weight fucoidan was obtained from *Sargassum siliquosum* with 107.3 kDa. However, in this present study, the obtained fucoidan was low molecular weight with 2–6 kDa from *Stoechospermum marginatum*.

The laminarin conjugated gold nanoparticles does not inhibit the Vero cells at higher concentration of 100 µg mL^−1^^79^. In another study, laminarin showed no cytotoxic effect on HDFa cell line (Normal primary dermal fibroblast) at 100 µg mL^−1^ concentration, and NHEK cell line (Primary normal human epidermal keratinocytes) at 500 µg mL^−1^ concentration^80^. Simultaneously, laminarin (100 µg mL^−1^) treated with human adipose stem cells and L929 cell line (Mouse fibroblast) exhibits no cytotoxic activity^81^. However, about 2 mg mL^−1^ concentration of laminarin for 24 h of treatment, selectively inhibited ovarian cancer (ES-2 and OV-90) and showed no cytotoxic effect on Zebrafish embryo xenograft model^9^. Moreover, the depolymerized low molecular weight laminarin enhanced the proliferation rate of normal fibroblasts, and eventually suppressed the melanomas by inducing TNF-α in a wound dressing study^82^. Relatively to the above studies, laminarin derived from *Padina pavonica* also showed no cytotoxic effects on Vero cells with an IC_50_ value of 123.54 ± 0.8 µg mL^−1^.

Fucoidan from *Cladosiphon okamurans* had no cytotoxic effect on Vero cells upto 2 mg mL^−1^ concentration, but inhibits Canine distemper virus (CDV) replication in Vero cells^83^. Concurrently, the same exhibit no cytotoxicity on Vero cells but inhibits the replication of Newcastle Disease Virus (NDV)^44^. In an intriguing study, fucoidan from *Turbinaria conoides* potentially inhibits cancer cells (A549—Human lung adenocarcinoma) by 25–75% in a dose dependent manner but doesn’t showed cytotoxicity on Vero cells^84^ and human skin keratinocytes (HaCaT cell line)^85^. This was again supported by another study, in which, fucoidan from *Fucus evanescens* possess no cytotoxic effect on Vero cells up to 2 mg mL^−1^ concentration^86^. Chantree et al.^87^ evidently reported that the fucoidan isolated from *Fucus vesiculosus* arrests cell cycle on Cholangiocarcinoma cells (CL-6) via apoptosis, whereas, the same fucoidan doesn’t exhibits any cytotoxic activity on OUMS cells (human embryonic fibroblast cell line). The present study also supports other studies by showing no cytotoxic effects on Vero cells.

Laminarin isolated from laminaria digitata enhances apoptosis in HT-29 colon cancer cells by inducing the sub-G1 and G2/M Phases^8^. Whereas, laminarin induces apoptosis through Fas and IGF-IR signaling pathways and ErbB (intrinsic apoptotic) pathways^88^. The four different seaweed extracts of *Sargassum latifolium* had shown IC_50_ values of 62.59 µg mL^−1^, 110.20 µg mL^−1^, 162.79 µg mL^−1^ and 51.15 µg mL^−1^ on HCT-116 Human colon cancer cells with promising anti-hypoxic activity^89^. However, laminarin from *Sargassum thunbergii* inhibited human lung adenocarcinoma (A549 cell line) with IC_50_ values of 2.70 mg mL^−1^ and 2.85 mg mL^−1^ for 12 and 24 h, respectively^90^. Intriguingly, different ranges of laminarin concentrations for 48 h significantly suppressed the cell viability and induces apoptosis in a dose-dependent manner on liver cancer cells (Bel-7404 and HepG2)^91^. In aggreement with Tian et al.^91^, our study resulted that the laminarin supplementation inhibits the cell viability of HT-29 Human colon cancer cells and which is proportional to the concentration of laminarin with an IC_50_ value of 57 ± 1.2 µg mL^−1^.

The fucoidan extract of *Sargassum horneri*^22^, *Chnoospora minima*^92^, *Ecklonia cava*^93^, *Laminaria japonica*, *Fucus vesiculosus*, and *Undaria pinnatifida*^94^, were found to suppress the proinflammatory cytokines such as IL-6, IL-1β, and TNF-α in vitro. Oral administration of fucoidan isolated from *Laminaria japonica* inhibits proinflammatory cytokines and enhanced antiinflammatory cytokines, respectively^95^ in vivo in cancer patients. Clinical trails on fucoidan from *Fucus vesiculosus* resulted that it downregulates the proinflammatory cytokines^96^. High molecular weight fucoidan from *Fucus vesiculosus* found to induce apoptosis by the activation of caspases in MCF-7 (human breast cancer) and HeLa (cervical cancer) in vitro^97^, and thus, fucoidan is a potential compound to treat melanoma including the health improvement of cancer patients in the stages of metastases^98^. The fucoidan extracted from *Sargassum cinereum* has an IC_50_ value of 250 µg mL^−1^ on Caco-2 human colorectal adenocarcinoma cells in vitro^37^. The IC_50_ values of Fucoidan isolated from the seaweed *Fucus vesiculosus* on DLBL (Diffuse Large B-cell Lymphoma) cell lines were 101.6 µg mL^−1^ on U2932 cell lines, 97.5 µg mL^−1^ on TMD8 cell lines, 93.7 µg mL^−1^ on NU-DUL-1 cell lines, 95.5 µg mL^−1^ on DB cell lines, 82.3 µg mL^−1^ on OCI-LY8 cell lines, and 80.0 µg mL^−1^ on SUDHL-4 cell lines^99^. Whereas, in this present study, the fucoidan extracted and purified from *Stoechospermum marginatum* has potential anticancer activity with an IC_50_ value of 24.4 ± 1.5 µg mL^−1^ on human liver cancer cells (Hep G2) in vitro. Simultaneously, fucoidan fraction derived from a brown seaweed *Hizikia fusiforme* was found inhibiting liver cancer cells (Hep3B) at a maximal inhibitory concentration of 33.53 ± µg mL^−1^^100^. Eventually, it has been resulted that, both the laminarin and fucoidan derived from PP and STM have no cytotoxic activity on Vero cells, but inhibited the proliferation cancer cells HT-29 (human colon cancer) and Hep G2 (human liver cancer).

Native laminarin induces apoptosis in LoVo (human metastatic colon carcinoma), whereas, enzyme-hydrolyzed laminarin also demonstrated potential anticancer activity over SK-MEL-28 (human melanoma), and DLD-1 (colon cancer) cell lines^101^. However, laminarin sulphate hampers heparanase activity in mouse melanoma cells (B16-BL6) and inhibits the metastasis of mammary adenocarcinoma^102^. Simultaneously, laminarin from *Dictyota dichotoma* inhibits matrix metalloproteinases MMP-2, MMP-9, and p-ERK1/2 signalling cascade and resulted in the suppression of progression and migration of human melanoma^103^. Increased concentration of cytoplasmic and mitochondrial calcium levels and induced apoptosis was seen in the ovarian cancer cells treated with the laminarin isolated from *Laminaria digitata*^9^. Similar kind of result was also reported in human colon cancer cells^18^.

Regular oral administration of fucoidan isolated from *Fucus evanescens* significantly enhances the antitumor, and anti-metastasis activity in mice model induced with Lewis lung adenocarcinoma^104^. Yang et al.^105^ recorded that the hydrolysed fucoidan had greater anticancer activity (> 75%) than the native fucoidan (37%) derived from *Undaria pinnatifida*. Comparatively, over sulphation of high molecular weight fucoidan > 30 kDa from *Undaria pinnatifida* had shown enhanced inhibition of cancer proliferation from 35 to 56%, whereas, over sulphation of low molecular weight fucoidan 5–30 kDa was found to inhibit from 37 to 68%^106^. The molecular sulphation of fucoidan plays a vital role in the function of fucoidan, hence, a decrease in the degree of sulfation in fucoidan (< 20%) drastically minimized the anticoagulant and anticancer efficacy of fucoidan^107^. Whereas, a high degree of sulfation induces anti-angiogenic activity and suppresses the growth of tumor cells^106,108^. Moreover, the sulphate groups in fucoidan have higher affinity and binding with the cationic proteins in the cancer cells and inhibit cancer proliferation^108,109^. Therefore, sulphate moiety is the most important factor in the function of fucoidan. In accordance with the above, the IC_50_ value of fucoidan on Hep G2 was found better than the same of laminarin with HT-29 due to the presence of sulphate group in fucoidan, which is advantageous for fucoidan (from STM) over laminarin (from PP) on the anticancer potential. The polysaccharides from seaweeds were reported with many beneficial activities. Further, fucoidan is of considerable interest in functional food, pharmaceutical, and cosmeceutical use^110^.

Fucoidan from seaweeds showed anticancer activity by enhancing the chromatin condensation, Bax, cleaved Cas-9 and poly-ADP ribose polymerase, and suppressed Bcl-2, p-PI3K, p-P38, p-Akt, and p-ERK genes in a dose-dependent manner in vitro in DU-145 prostate cancer cells^111^. Similarly, fucoidan hampers PI3K, ERK, and MAPK pathway and thereby suppressing the cancer progression and also inhibits the expression of Bcl-2, Bax, and enhanced caspase-dependent apoptosis in LM3 (Murine mammary adenocarcinoma) and BEL-7402 (hepatocellular carcinoma) cell lines^112^. Fucoidan from *H. fusiforme* upregulated Bcl-2 associated X protein, caspase-3, and induced mitochondria mediated apoptosis in human liver cancer cells^100^. The natural killer (NK) cells followed by IL-2 and IFN-γ were induced in mice model fed with fucoidan orally in tumour induced mice than the control mice^113–116^. About 1 mg mL^−1^ concentration of fucoidan from *Cladosiphon okamuranus* to Huh7 liver cancer cells had increased the G0/G1 phase population, and decreased S-phase population and resulted that the fucoidan arrests cell cycle at G0/G1 phase^117^.

AO/EB assay is a simple and cost-effective assay to detect apoptosis^47^. When live cells are stained with acridine orange emits green fluorescence, whereas, dead cells emit orange-red by ethidium bromide due to the loss of cytoplasmic membrane of dead cells and interaction between ethidium bromide and the intercalating DNA^118^. Therefore, this method is effective in identifying even mild nucleic acid damages^119^. However, bright-green fluorescence illustrates early apoptotic phase when fragmented chromatin structures stained. Orange-red fluorescence resulted in the late apoptotic phase, when ethidium bromide stained with nuclei^120^. Concurrently, in the present investigation, both the cancer cell lines HT-29 (colon), and Hep G2 (liver) had undergone apoptotic and necrotic cell death phase and were evidently confirmed by AO/EB staining assay under fluorescent microscopy^121,122^. The fragmented DNA was visually seen as orange and red fluorescence in both the laminarin and fucoidan treated cancer cells.

Unlike programmed cell death (apoptosis), necroptosis is a passive cell death by the uncontrolled expression of inflammatory factors. Thus, necrosis deals with the immunological concept of cell cycle arrest and death^123^. Moreover, cancer cells progressively develop resistant to some cancer drugs by deletion in specific genes, genetic aberration, and overexpression of anti-apoptotic proteins which is a major defect in the apoptotic death machinery^124–126^. Therefore, programmed necrotic cell death is an alternate approach in anticancer treatment by inducing the immune system. However, in the current investigation, based the DNA fragmentation assay, it was evidently clear that the laminarin and fucoidan from PP and STM showed anticancer activity by forming DNA smear on both colon (HT-29) and liver (Hep G2) cancer cell lines, respectively by inducing the necrotic factors based on Zhivotosky and Orrenius^127^. Based on the present investigation, laminarin and fucoidan are the potential bioactive compounds for the treatment of cancer.

## Conclusion

In the present study, both laminarin and fucoidan were isolated from *Padina pavonica* (PP), and *Stoechospermum marginatum* (STM). Laminarin constitutes glucose, and mannose, whereas, fucoidan is rich in fucose, galactose, and xylose with sulphate groups. However, the molecular weight was determined between 3 and 5 kDa for laminarin and 2 kDa, and 6 kDa for fucoidan. Both the polysaccharides showed no cytotoxicity on Vero cells, but laminarin inhibits the proliferation of human colon cancer cells (HT-29) in vitro with an IC_50_ value of 57 ± 1.2 µg mL^−1^. Simultaneously, fucoidan also suppresses the progression of human liver cancer cells (Hep G2) in vitro with an IC_50_ value of 24.4 ± 1.5 µg mL^−1^. The fucoidan activity was found relatively greater than laminarin may be due to the presence of sulphate groups in it. Moreover, necrosis based cancer cell death was determined by fragmented DNA smear in both the cases. Therefore, it has been concluded that both the laminarin from PP and fucoidan from STM have potential anticancer activity on human colon cancer and liver cancer cells. Additionally, molecular marker interventions are required for future investigations.

### Supplementary Information

Supplementary Information 1.

Supplementary Information 2.

Supplementary Information 3.

Supplementary Information 4.

## Data Availability

All the data are available with Prof. Elumalai Sanniyasi (ananandal67@gmail.com).
