# Supplementary figures and images for: In vitro anticancer potential of laminarin and fucoidan from Brown seaweeds
Source: Sci Rep. 2023 Sep 2;13:14452. doi: 10.1038/s41598-023-41327-7 (PMC10475116; doi:10.1038/s41598-023-41327-7)

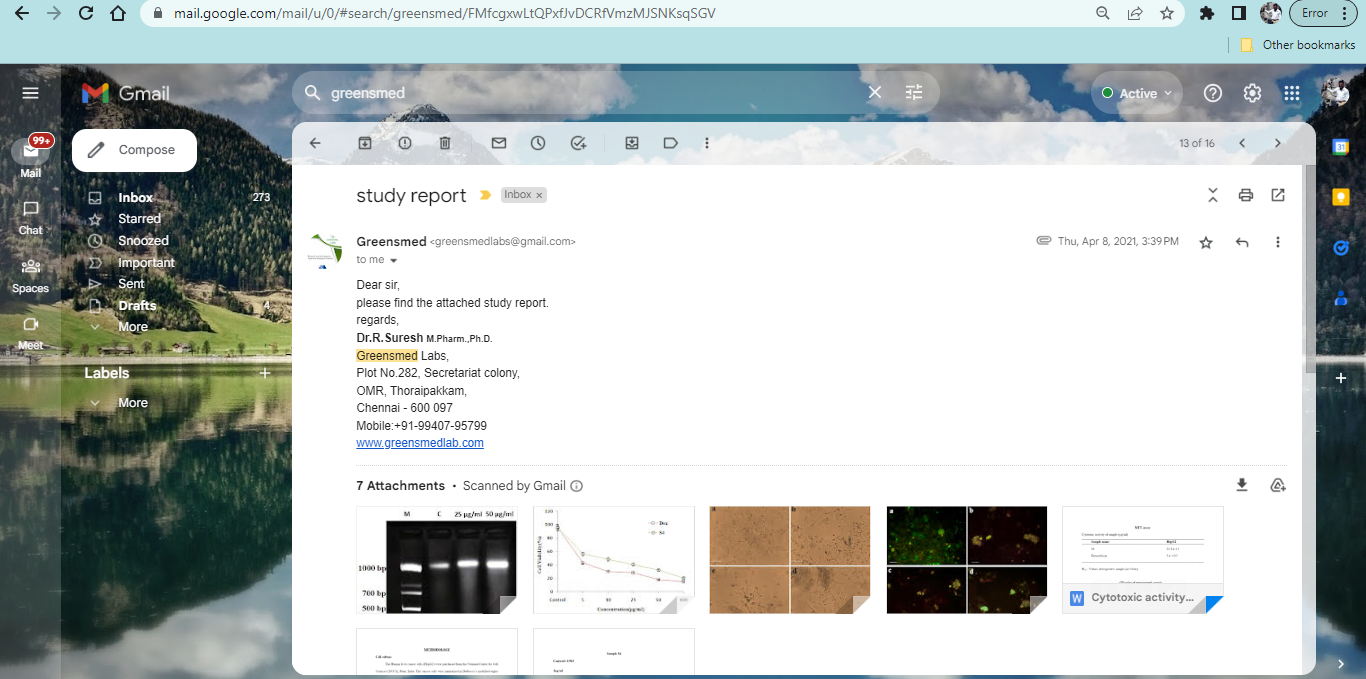

Supplement: Supplementary file 1 — Supplementary Information 1. [file 41598_2023_41327_MOESM1_ESM.bmp]

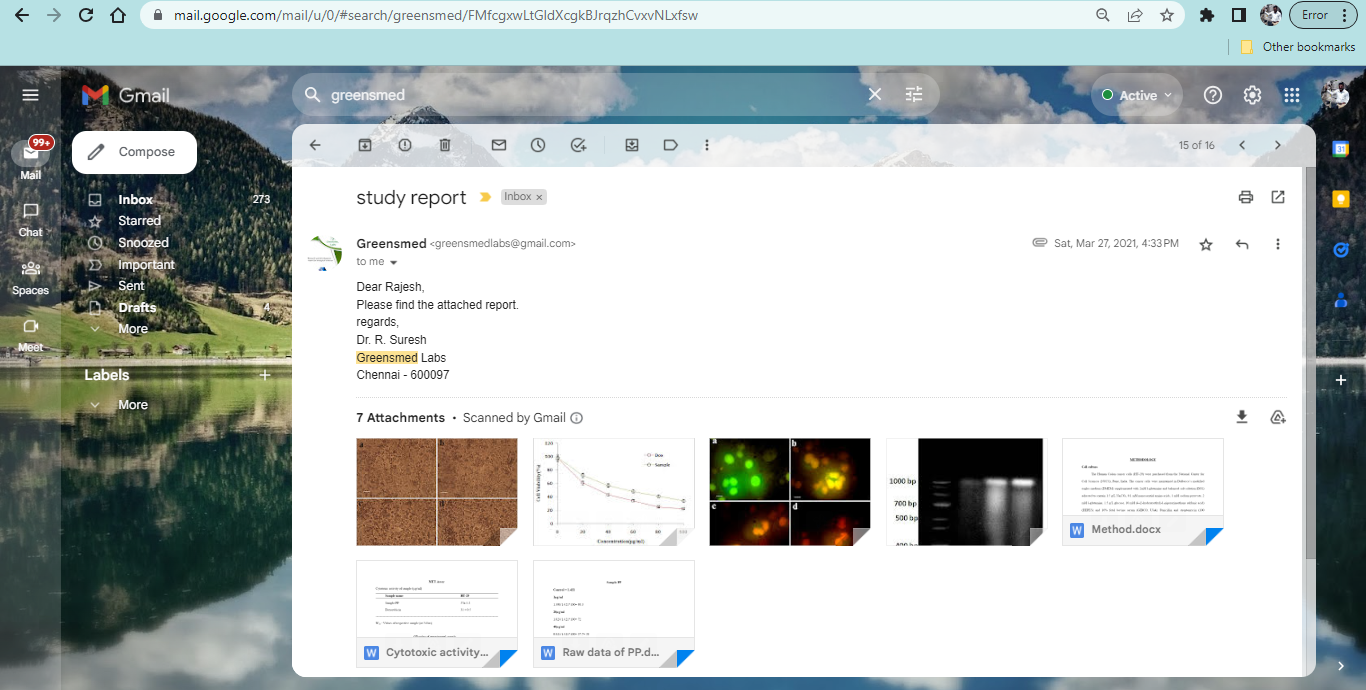

Supplement: Supplementary file 2 — Supplementary Information 2. [file 41598_2023_41327_MOESM2_ESM.bmp]

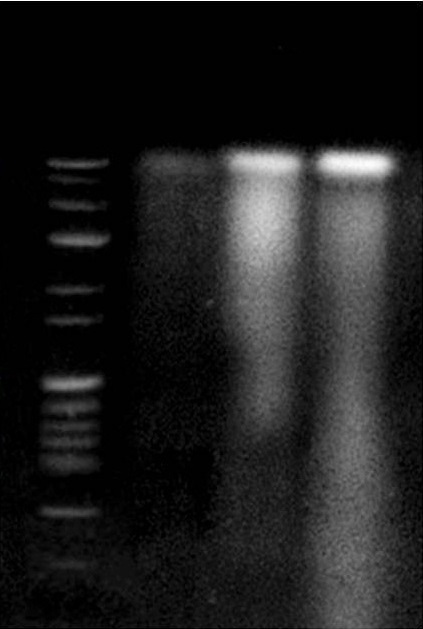

Supplement: Supplementary file 3 — Supplementary Information 3. [file 41598_2023_41327_MOESM3_ESM.jpg]

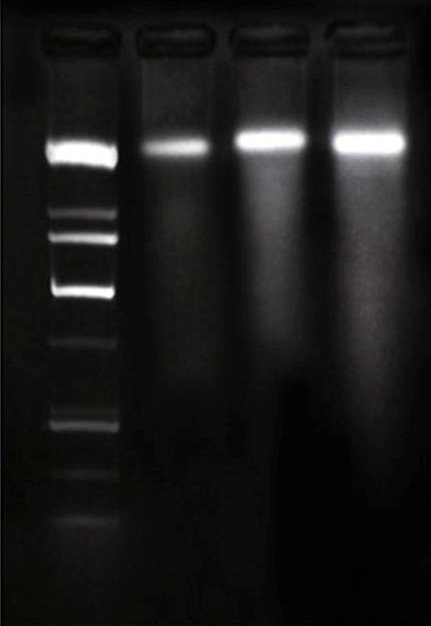

Supplement: Supplementary file 4 — Supplementary Information 4. [file 41598_2023_41327_MOESM4_ESM.jpg]
